# Anti-Inflammatory, Antioxidative, and Hepatoprotective Effects of Trans Δ9-Tetrahydrocannabinol/Sesame Oil on Adjuvant-Induced Arthritis in Rats

**DOI:** 10.1155/2018/9365464

**Published:** 2018-06-25

**Authors:** Morouj Ismail, Hiba Hasan, Youmna El-Orfali, Hanan Ismail, Ghada Khawaja

**Affiliations:** Department of Biological Sciences, Faculty of Science, Beirut Arab University, Lebanon

## Abstract

Rheumatoid arthritis (RA) is a painful chronic autoimmune disease affecting the joints. Its first-line therapy, Methotrexate (MTX), although effective in ameliorating the progress of the disease, induces hepatotoxicity over long-term usage. Thus, seeking natural compounds with fewer side effects could be an alternative therapeutic approach. This study aimed to investigate the anti-inflammatory, antiarthritic, and antioxidative effects of synthetic* trans*-Δ9-tetrahydrocannabinol (Δ9-THC) dissolved in sesame oil (Dronabinol) against MTX in adjuvant-induced arthritis (AIA) rat model. Daily oral administration of Δ9-THC/sesame oil, over a period of 21 days, was well tolerated in arthritic rats with no particular psychoactive side effects. It markedly attenuated the severity of clinical manifestations, recovered the histopathological changes in tibiotarsal joints, and repressed the splenomegaly in arthritic rats. Δ9-THC/sesame oil therapy showed similar effects to MTX in neutralizing the inflammatory process of AIA, through attenuating erythrocyte sedimentation rate (ESR) scores and proinflammatory cytokines, including tumor necrosis factor-alpha (TNF-*α*), interleukin 1-beta (IL-1*β*), and interleukin-6 (IL-6) levels, to normal values. As opposed to MTX, this natural combination markedly protected the liver of arthritic rats and downregulated the induced oxidative stress by increasing the antioxidant defense system such as activities of catalase and superoxide dismutase (SOD) and levels of glutathione (GSH). These results suggest promising effects for the clinical use of Δ9-THC/sesame oil therapy in alleviating arthritic clinical signs as well as arthritis-induced liver injury.

## 1. Introduction

Rheumatoid arthritis (RA) is a chronic inflammatory autoimmune disease that attacks the synovial lining of diarthrodial joints and causes formation of pannus tissue. This tissue is known to expand and invade the cartilage and bone, eventually damaging them and leading to physical disabilities such as the loss of articular function [1, 2]. One of the most important mediators for the pathogenesis of RA are proinflammatory cytokines, specifically tumor necrosis factor-alpha (TNF-*α*), interleukin 1-beta (IL-1*β*), and interleukin 6 (IL-6) [3, 4]. Besides having local effects on the synovial microenvironment where they are released, these cytokines can also reach systemic circulation and mediate inflammatory responses in other organs. Moreover, they stimulate phagocytic cells to produce reactive oxygen species (ROS), which mediate tissue injury in RA. Excessive production of free radicals in arthritic animals and RA patients is associated with a stimulated prooxidant system and a deficient antioxidative defense system, thus leading to oxidative stress and lipid peroxidation that damage not only the synovium, but also other organs such as the liver [5–11].

The therapeutic management of RA involves synthetic drugs that meet the disease by reducing pain, swelling, and symptoms and delaying the disease progression. These drugs include nonsteroidal anti-inflammatory drugs (NSAIDS), disease modifying antirheumatic drugs (DMARDs), and corticosteroids [1]. Among DMARDs, Methotrexate (MTX) is the most common conventional therapeutic agent for the disease. However, many RA cases were unresponsive to this drug [12]. Moreover, it is associated with several side effects, most commonly hepatotoxicity, pancytopenia, acute renal failure, pneumonitis, and other complications [13].

Cannabinoids are gaining much interest as potential therapeutic agents for many diseases due to their anti-inflammatory and anticancer effects. They are classified into 3 classes: phytocannabinoids, synthetic cannabinoids, and endocannabinoids. Phytocannabinoids constitute the class of naturally occurring cannabis derived from the plant* Cannabis sativa *and including more than 60 compounds [14]. The first discovered and tested cannabinoid was* trans*-Δ9-tetrahydrocannabinol (Δ9-THC). Several other compounds were isolated such as cannabinol, cannabidiol, and cannabicyclol. Endocannabinoids include endogenous ligands that were discovered later, such as anandamide, 2-arachidonoyl glycerol (2-AG), 2-arachidonyl glyceryl ether (2-AGE), and* N*-arachidonoyl dopamine (NADA) [15]. These are all derivatives of the parent compound arachidonic acid, which is an important lipid-signaling molecule and a key component of the inflammatory pathway [16].

The action of cannabinoids depends on the receptor they interact with. The first two discovered receptors were cannabinoid receptors 1 and 2: CB1 and CB2. CB1 receptors are found throughout the brain, spleen, eye, testis, and uterus. CB2 receptors are associated with the cells and organs of the immune system, as well as tumor cells. CB1 binding mediates the psychoactive properties of the cannabinoids while CB2 mediates their immune-regulatory effects [17, 18]. Preclinical and clinical studies using cannabis-based therapy have demonstrated anti-inflammatory effects of these compounds. The dimethylheptyl homologue of the natural* C. sativa *plant product THC-11-oic acid, known as ajulemic acid, had a suppressive effect on joint inflammation in adjuvant-induced arthritic (AIA) rats with fewer side effects as compared to common nonsteroidal anti-inflammatory drugs [19]. Moreover, cannabidiol, a nonpsychoactive cannabinoid, showed potential anti-inflammatory and immunosuppressive effects on collagen-induced arthritic rats [20–22]. As for Δ9-THC, it was shown to exhibit analgesic effect in chronic pain patients and multiple sclerosis (MS) cases [23–25].

On the other hand, sesame oil, derived from the plant species* Sesamum indicum *L and rich in sesamol, sesamin, and other lignans, has proven to possess a potential antioxidative effect [26, 27] and credits in alleviating hepatotoxicity specifically [28, 29]. Moreover, in an experimentally induced arthritic rat model, sesame oil was recently shown to exhibit an antiarthritic effect [27].

Therefore, we aimed in the present study to investigate the potential therapeutic effects of Dronabinol, a synthetic Δ9-THC prepared in sesame oil, against the standard drug MTX, on a well-known experimental model of RA. We focused here on assessing the anti-inflammatory, antiarthritic, and antioxidative activities of Dronabinol against adjuvant-induced arthritis in rats.

## 2. Materials and Methods

### 2.1. Animals and Experimental Design

Forty healthy male Sprague Dawley rats (180-220g) were obtained from the animal house facility at the Faculty of Science, Beirut Arab University, Lebanon. Rats were maintained at a constant temperature of 22°C under a 12-h light/dark cycle and fed by a standard pellet diet and water ad libitum. Animal experimentation was approved and performed in accordance with the guidelines for animal care issued by the institutional review board (IRB) at Beirut Arab University (Approval code: 2017A-0030-S-P-0214). Adjuvant arthritis was induced by subplantar injection into the left hind paw of 0.1 ml of complete Freund's adjuvant (CFA) consisting of heat-killed* Mycobacterium tuberculosis* (strain H37Ra) (1mg/ml) in paraffin oil and mannide monooleate (InvivoGen, USA). The first immunization was then followed by two booster intradermal injections of the same dose in the base of the tail, as previously described [30]. To reduce any discomfort caused by CFA injection, rats were gently restrained using a DecapiCone, which allowed for the proper delivery of CFA. Rats were monitored for the onset of arthritis as characterized by erythema in the injected paw. Two days following CFA challenge, rats were randomly assigned into four AIA groups where each group consisted of eight rats (n=8): untreated control AIA group; AIA group treated with MTX (0.75mg/kg/week) for 21 days, intraperitoneally (Sigma-Aldrich Co, USA) [30]; AIA group treated with Dronabinol (Δ9-THC/sesame oil, 2.5mg/kg/daily) for 21 days, by diet (Dronabinol, Watson Laboratories, USA); and AIA group treated with equivalent volumes of sesame oil for 21 days, by diet. Dose of Δ9-THC was determined according to a study conducted in collagen-induced arthritis model wherein several doses of a cannabinoid, cannabidiol, were tested [20]. For AIA groups receiving treatment by diet, the drug was soaked into crackers before being delivered to rats. A normal healthy group of eight rats was injected with saline instead of CFA and followed up in parallel. On day 23, rats were anesthetized and sacrificed by cervical dislocation. Whole blood samples were withdrawn from the heart by cardiac puncture, left hind paws were excised, and autopsy liver samples were also collected, frozen in liquid nitrogen, and stored at -80°C.

### 2.2. Assessment of Disease Progression

The body weight of normal and arthritic rats was monitored every two days throughout the entire experiment. The severity of arthritis was assessed on days 2, 8, 16, and 22. It was graded on a scale of 0-4 for the hind paw according to the following criteria: 0: no signs of erythema and swelling, 1: erythema and mild swelling confined to the tarsals or ankle joints, 2: erythema and mild swelling extending from the ankle to the tarsals, 3: erythema and moderate swelling extending from the ankle to metatarsal joints, 4: erythema and severe arthritis encompassing the ankle, foot, and digits [31]. After sacrifice, the spleen was collected and weighed. The spleen index was measured as the ratio of spleen net weight over body net weight and expressed in mg/g [32].

### 2.3. Histopathological Examination of Tibiotarsal Joint and Liver

Left hind paws were excised and fixed in 10% neutral buffered formalin for 48 hours. They were then decalcified in 10% formic acid and 8% hydrochloric acid for 2 weeks, embedded in paraffin, and cut into 4 *μ*m thick sections. The sections were stained with haematoxylin and eosin (H&E) staining and examined microscopically. The histological changes typically occurring in arthritic rats—synovial hyperplasia, cellular infiltration, and pannus formation—were graded by two independent blind observers [33]. Joint sections were also stained by Masson's Trichrome staining to assess the extent of cartilage erosion by scoring the presence of calcified cartilage [33].

Livers were also excised and processed for H&E staining. The pathological changes occurring—lobular and portal inflammatory infiltration, vascular congestion, and multinucleation—were examined by a pathologist blinded to the treatment. Scoring of lobular and portal inflammation was done as follows: + minimal; ++ mild changes; +++ moderate changes, and ++++ severe changes.

### 2.4. Hematological and Serological Assays

ESR, as a marker of inflammation, was determined for all groups on day 23 using modified Westergren method [34]. Levels of IL-6, IL-1*β*, and TNF-*α* were measured in rat sera using commercially available ELISA kits (Research and Development (R&D) Systems, Minneapolis). Activities of liver enzymes, alanine transaminase (ALT) and aspartate transaminase (AST), were measured in the sera of all groups using ALT (Analyticon, Biotechnologies) and AST (Spinreact) kits, respectively. All assays were performed according to the manufacturer's instructions.

### 2.5. Total Protein Quantification in Liver Samples

Autopsy liver samples were homogenized (10% w/v liver tissue in 1.15M KCL buffer) using Teflon Potter-Elvehjem homogenizer, and total protein content was determined according to Bradford's method [35] and was standardized using a BSA standard curve. Protein concentration in liver homogenates was expressed in mg/ml and was used to express the results of the following assays.

### 2.6. Determination of Lipid Peroxidation Levels in Liver Homogenates

As a biomarker of oxidative stress, the extent of lipid peroxidation in liver homogenates was assessed by measuring the formation of malondialdehyde (MDA) using the thiobarbituric acid-reactive substances assay (TBARS) [36]. Briefly, to 500 *μ*l of 10% w/v liver homogenate prepared in 1.5M KCL buffer, 1 ml of TBA:TCA:HCl reagent (0.38% thiobarbituric acid, 15% trichloroacetic acid, and 0.25 N hydrochloric acid, ratio 1:1:1) was added, boiled for 15 min, and cooled. After centrifugation, absorbance of TBARS was measured at 532 nm against a blank. The MDA content was calculated as nmol of TBARS per mg of protein.

### 2.7. Determination of Antioxidant Defense Biomarkers in Liver Homogenates

The activity of catalase was determined in 10% liver homogenates prepared in 0.05M phosphate buffer as described by Weydert and Cullen [37]. It was estimated by measuring, spectrophotometrically at 240 nm, the decrease in hydrogen peroxide (H_2_O_2_) concentration over time. Catalase activity was expressed as mmol per minute per mg of protein.

Activity of superoxide dismutase (SOD) was assessed in 10% liver homogenates prepared in 50 mM sodium phosphate buffer (pH 7.8) following the protocol of Beauchamp and Fridovich [38]. Based on the inhibition of nitroblue tetrazolium (NBT) reduction to a water-soluble formazan dye, SOD inhibition activity was determined spectrophotometrically at 560 nm and expressed as units per mg of total protein.

The concentration of glutathione GSH was also determined in 10% liver homogenate prepared in 5% metaphosphoric acid and 0.6% sulfosalicylic acid as described by Rahman and Biswas [39]. GSH content was assayed using a kinetic assay in which catalytic amounts of GSH cause a continuous reduction of 5,5′-dithiobis-(2-nitrobenzoic) acid (DTNB) to form a yellow product 5′-thio-2-nitrobenzoic acid (TNB). Absorbance was read at 412 nm, and GSH concentration was calculated from a standard curve and expressed as *μ*M or nM per mg of protein.

### 2.8. Data Analysis

Two-way analysis of variance (ANOVA) was used for statistical analysis of mean macroscopic changes between groups. For all other assays, one-way ANOVA was used to determine the significance between groups followed by Tukey-Kramer correction for multiple comparisons. Analysis of data and presentation of graphs were performed using GraphPad Prism software version 7.00 (San Diego, CA). P values ≤ 0.05 were considered statistically significant.

## 3. Results

### 3.1. Δ9-THC/Sesame Oil Ameliorates Disease Severity in AIA Rat Model

To evaluate the antiarthritic effect of Δ9-THC/sesame oil in AIA rats, we first studied its influence on AIA clinical manifestations by monitoring the arthritic macroscopic score and body weight once per week and by examining the spleen index at the end of the experiment. MTX, being one of the most prevalent DMARDs for RA treatment, was used as a positive control.

**Figure 1:**
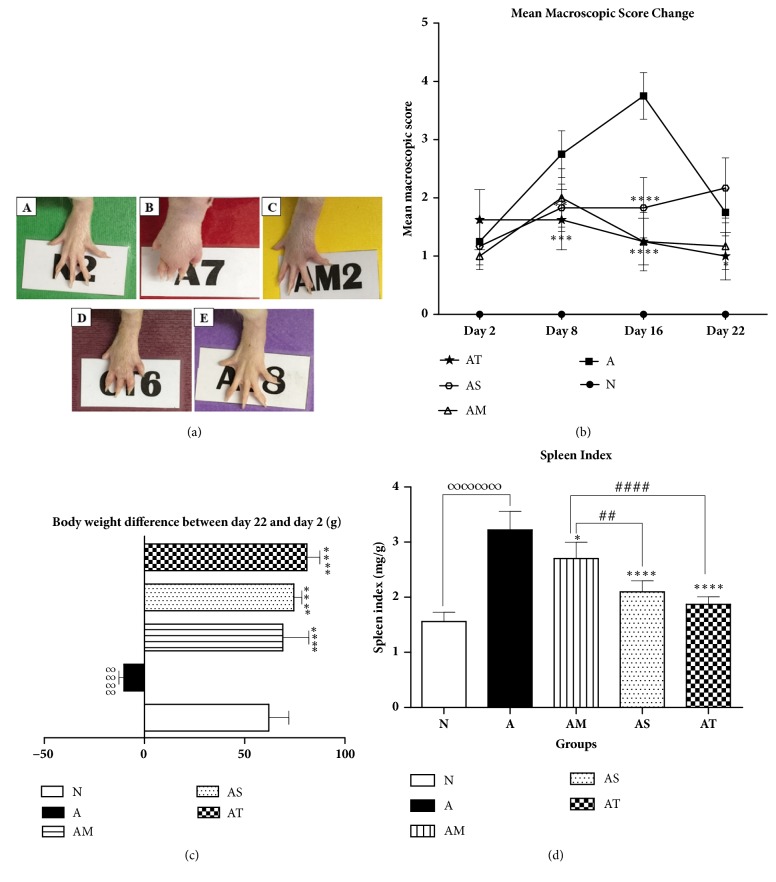
**Effect of treatments on left hind paw, body weight, and spleen index**. (a) Macroscopic image of left hind paw at day 16 in following groups: (A) normal [N], (B) untreated AIA [A], (C) AIA treated with MTX [AM], (D) AIA treated with sesame oil [AS], and (E) AIA treated with Δ9-THC/sesame oil [AT]. (b) Mean macroscopic scores change, (c) body weight difference, and (d) spleen index among groups. All values are presented as mean ± SD of 8 rats per group. ∞∞∞∞p<0.0001 for A compared with N. *∗*p<0.05, *∗∗*p<0.01, *∗∗∗*p<0.001, *∗∗∗∗*p<0.0001 compared with A. ##p<0.01 and ####p<0.0001 compared with the positive control group AM.

As shown in Figure 1(b), swelling and inflammation in CFA injected hind paws started to manifest themselves 24 hours after CFA challenge in all AIA groups when compared to normal healthy group. The severity of arthritis was evaluated by using a macroscopic scoring system ranging from 0 to 4. Untreated AIA control rats showed a gradual increase in mean macroscopic scores with a peak of score 4 on day 16 after challenge, followed by a decrease to score 2 at the end of the follow-up (Figure 1(b)). The tibiotarsal joints of these arthritic rats showed massive swelling and erythema at day 16 when compared to the normal ones (Figure 1(a)). Treatment with sesame oil alone significantly reduced the paw swelling and arthritis scores (mean scores 2, day 16) when compared to untreated AIA group, but this effect was not sustained till the end of the experiment. In contrast, as compared to AIA group, Δ9-THC/sesame oil treatment suppressed the paw swelling induced in AIA rats and significantly diminished the arthritic scores from day 8 till day 22 (mean scores 1 at day 16 and 22) in a similar manner to MTX treatment (Figures 1(a) and 1(b)).

On the other hand, the growth of arthritic rats was found to be impeded as they showed a significant reduction in body weight throughout the experiment when compared to normal rats, indicating the severity of arthritis disease. However, all treatments resulted in a significant increase in body weight as compared to AIA control group (Figure 1(c)). Interestingly, Δ9-THC/sesame oil- and sesame oil-treated rats significantly gained more weight than other groups and their growth was comparable to normal healthy group.

Furthermore, compared to normal healthy rats, arthritic rats showed a marked increase in the spleen index, indicating a splenomegaly. However, arthritic groups treated with MTX, sesame oil, and Δ9-THC/sesame oil showed an approximately 16, 35, and 46% decrease in spleen index when compared to AIA untreated group, respectively (Figure 1(d)). The spleen indices of Δ9-THC/sesame oil-treated group and sesame oil-treated group were significantly lower than that of MTX-treated group (with p<0.0001 and p<0.01, respectively) and comparable to that of the normal group.

### 3.2. Δ9-THC/Sesame Oil Ameliorates AIA-Associated Histopathological Changes in Tibiotarsal Joints

**Figure 2:**
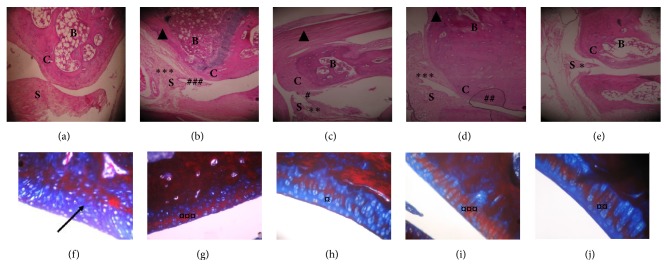
**Light micrographs of tibiotarsal joint sections of rat left hind paws**. (a, b, c, d, e) H&E stained micrographs. (a) Normal rat shows normal appearance of the synovial membrane and cartilage. (b) AIA untreated rat shows severe synovial hyperplasia (*∗∗∗*) with cellular infiltration (###). (c) MTX-treated AIA rat shows moderate synovial hyperplasia (*∗∗*) and minimal infiltration (#). (d) Sesame oil-treated AIA rat shows severe synovial hyperplasia (*∗∗∗*) with some infiltration (##). (e) Δ9-THC/sesame oil-treated AIA rat shows minimal synovial hyperplasia (*∗*) with no infiltration. B: bone, C: cartilage, S: synovium. (f, g, h, i, j) Masson's Trichrome stained micrographs. (f) Normal rat shows normal and intact cartilage. (g) AIA untreated rat shows severe cartilage calcification (*¤¤¤*). (h) MTX-treated AIA rat shows minimal cartilage calcification (*¤*). (i) Sesame oil-treated AIA rat shows severe cartilage calcification (*¤¤¤*). (e) Δ9-THC/sesame oil-treated AIA rat shows moderate cartilage calcification (*¤¤*). (Original magnification x40.)

To assess whether Δ9-THC/sesame oil treatment can recover the histopathological changes associated with AIA, H&E and Masson Trichrome staining of tibiotarsal joint sections were performed at the end of the experiment (day 23). The histological examination revealed significant differences among the five groups. The normal group showed normal architecture of the joint with a preserved synovium, absence of inflammation, absence of pannus tissue, a well-defined joint space (Figure 2(a)), and presence of intact cartilage (blue staining, Figure 2(f)). However, untreated AIA group presented severe synovial hyperplasia and prominent cellular infiltration with pannus formation (Figure 2(b)). Moreover, arthritic rats showed an extensive presence of calcified cartilage stained in red (Figure 2(g)), reflecting the severity of cartilage erosion. Treatment with Δ9-THC/sesame oil exhibited advantageous effects on these pathological manifestations. It reduced the severity of synovial hyperplasia and cellular infiltration to a minimal grade (Figure 2(e)) and cartilage calcification to a moderate level (Figure 2(j)) and restored as well a normal joint space similar to the positive control group (MTX-treated group, Figures 2(c) and 2(h)), whereas treatment with sesame oil did not ensue any significant improvement in the joint architecture when compared to untreated AIA group (Figures 2(d) and 2(i)).

### 3.3. Δ9-THC/Sesame Oil Exhibits a Potent Anti-Inflammatory Effect in AIA Rat Model

**Figure 3:**
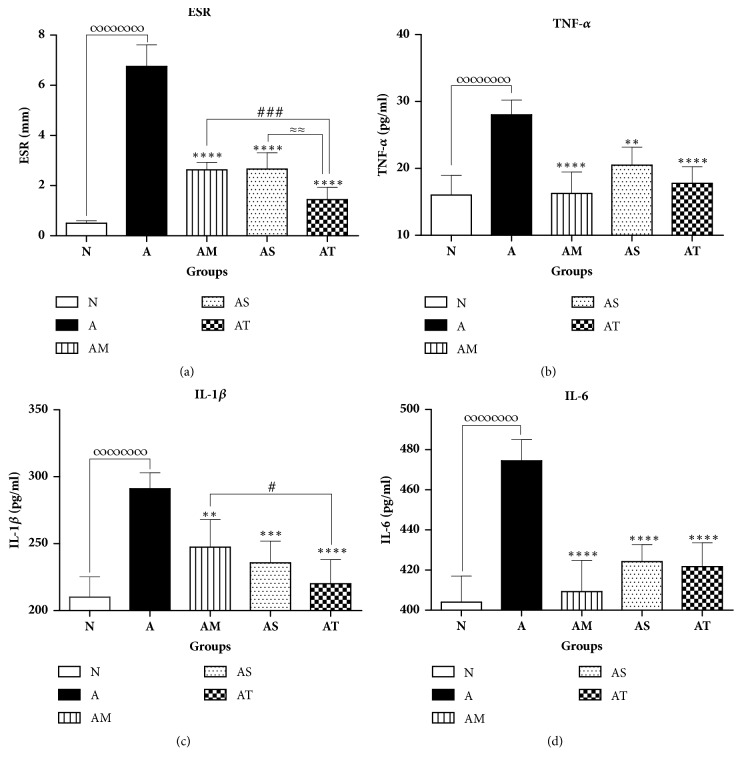
**Effect of treatments on inflammatory markers**. (a) Blood ESR, serum, (b) TNF-*α*, (c) IL-1*β*, and (d) IL-6 among groups. All values are presented as mean ± SD of 8 rats per group. ∞∞∞∞p<0.0001 for A compared with N. *∗∗*p<0.01, *∗∗∗*p<0.001, *∗∗∗∗*p<0.0001 compared with arthritic group A. #p<0.05, ###p<0.001 compared with positive control group AM. ≈≈p<0.01 between AS and AT.

To better appraise the anti-inflammatory effect of Δ9-THC/sesame oil in AIA model, blood ESR and serum proinflammatory cytokines levels (TNF-*α*, IL-1*β*, and IL-6) were determined at day 23. As shown in Figure 3(a), mean ESR scores significantly increased by 93% in untreated AIA group versus normal group, reflecting the occurrence of systemic inflammatory response and the production of acute phase proteins in arthritic rats. Besides, TNF-*α*, IL-1*β*, and IL-6, known to be primarily involved in RA pathogenesis, were found to be significantly upregulated in untreated AIA group as compared to the normal group (Figures 3(b), 3(c), and 3(d)).

In contrast, a significant reduction in all inflammatory markers was observed in all treated AIA groups compared with the untreated AIA group. Indeed, treatment with MTX, sesame oil, and Δ9-THC/sesame oil diminished mean ESR scores by 61%, 60%, and 79% relative to untreated AIA group, respectively (Figure 3(a)). More importantly, mean ESRs scores in Δ9-THC/sesame oil-treated group were significantly lower than MTX-treated group, with no statistical difference when compared to the normal group.

As for proinflammatory cytokines, mean values of TNF-*α*, IL-1*β*, and IL-6 were found to be significantly reduced in MTX-, sesame oil-, and Δ9-THC/sesame oil-treated AIA groups when compared to the untreated AIA group, with no statistical difference relative to the normal one (Figures 3(b), 3(c), and 3(d)). Overall, Δ9-THC/sesame oil treatment exhibited slightly better anti-inflammatory effect than sesame oil treatment and a comparable effect to the standard MTX treatment used as a positive control.

### 3.4. Δ9-THC/Sesame Oil Attenuates the Systemic Inflammation Induced in Liver of AIA Rats

**Figure 4:**
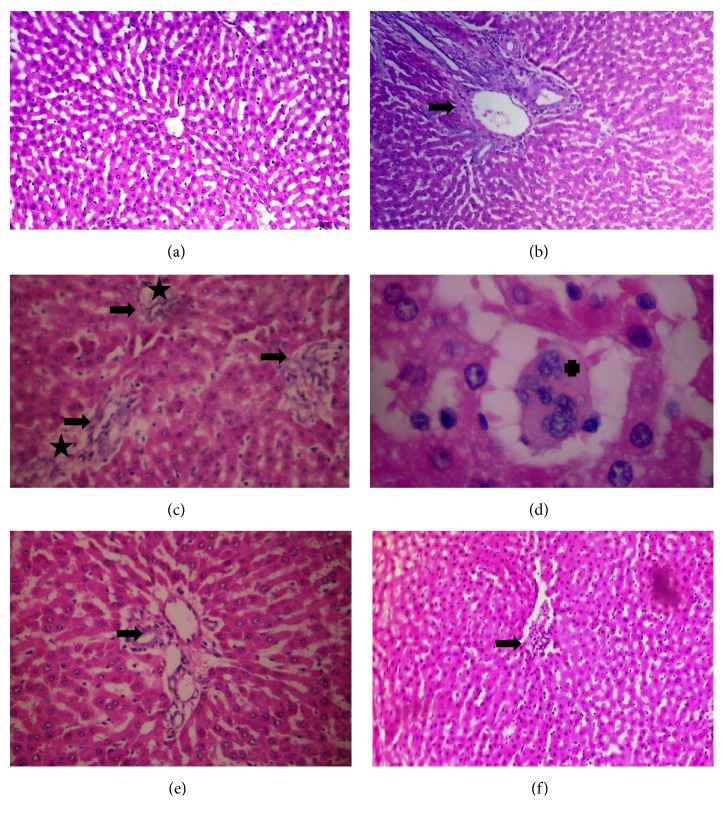
**Effect of treatments on liver histology**. Photomicrographs of liver sections stained by H&E. (a) Normal rat showing normal liver architecture. (b) AIA untreated rat showing moderate portal inflammation (+++) and neutrophil infiltration (black arrow). (c, d) MTX-treated AIA rat shows distortion of liver architecture with severe portal inflammation (++++) and neutrophil infiltration (black arrow), vascular congestion (aster), and multinucleation (cross). (e) Sesame oil-treated AIA rat shows mild portal inflammation (++) with neutrophil infiltration (arrow). (f) Δ9-THC/sesame oil-treated AIA rat shows minimal portal inflammation (+) with neutrophil infiltration (arrow) and normal liver architecture.

To further investigate the effect of Δ9-THC/sesame oil on systemic inflammation induced in AIA rat model, histological examination of liver sections of all groups was performed at the end of the follow-up. Untreated AIA group exhibited moderate portal inflammation and neutrophil infiltration (Figure 4(b)) compared to the normal group that has normal liver architecture (Figure 4(a)). However, in MTX-treated group, this architecture was distorted with obvious markers for liver damage including severe portal inflammation, neutrophil infiltration, vascular congestion, and multinucleation (Figures 4(c) and 4(d)). More importantly, the severity of portal inflammation and neutrophil infiltration decreased remarkably in sesame oil-treated and Δ9-THC/sesame oil-treated AIA groups from mild to minimal, respectively, with no evidence of vascular congestion or multinucleation (Figures 4(e) and 4(f)).

**Figure 5:**
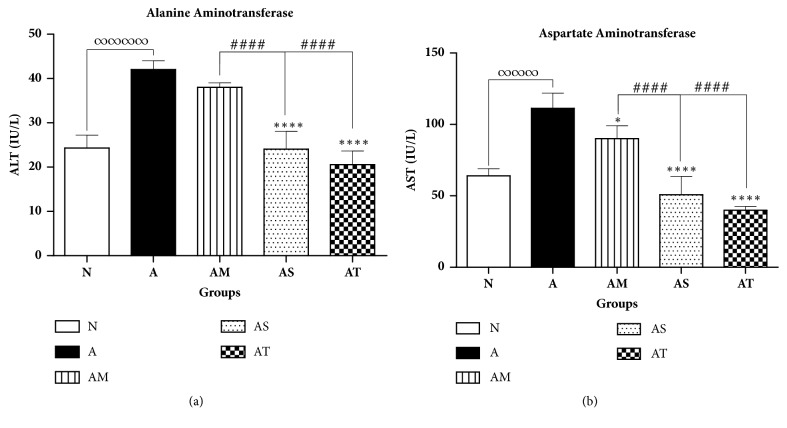
**Effect of treatments on liver function enzymes**. (a) ALT and (b) AST serum activity levels among groups. All values are presented as mean ± SD of 8 rats per group. ∞∞∞∞p<0.001 for A compared with N. *∗*p<0.05, *∗∗∗∗*p<0.0001 compared with arthritic group A. ####p<0.0001 compared with positive control AM.

To further investigate the effect of Δ9-THC/sesame oil on liver inflammation, the activities of liver function enzymes, ALT and AST, were determined by measuring their levels in the sera of all rats on day 23. ALT and AST are normally found in blood at low activity levels. Elevation in serum activity levels of these enzymes may reflect inflammation or liver injury. Untreated AIA group exhibited significantly higher activity levels of ALT and AST as compared to normal group. Similarly, MTX-treated group showed a significant elevation in the activity levels of these enzymes when compared to normal values. However, compared to untreated AIA group, activities of ALT and AST decreased significantly in AIA groups treated with sesame oil (by 43% and 54%, respectively) and in Δ9-THC/sesame oil-treated group (by 51% and 64%, respectively) with no statistical difference relative to normal group (Figures 5(a) and 5(b)).

### 3.5. Δ9-THC/Sesame Oil Diminishes the Oxidative Stress and Upregulates the Antioxidative Defense System in the Liver of AIA Rats

To examine whether Δ9-THC/sesame oil can attenuate the oxidative stress known to be involved in the pathogenesis of arthritis, levels of MDA, a major biomarker of lipid peroxidation and activities of antioxidant enzymes such as SOD and catalase as well as concentration of nonenzymatic antioxidant GSH, were determined in liver homogenates.

**Figure 6:**
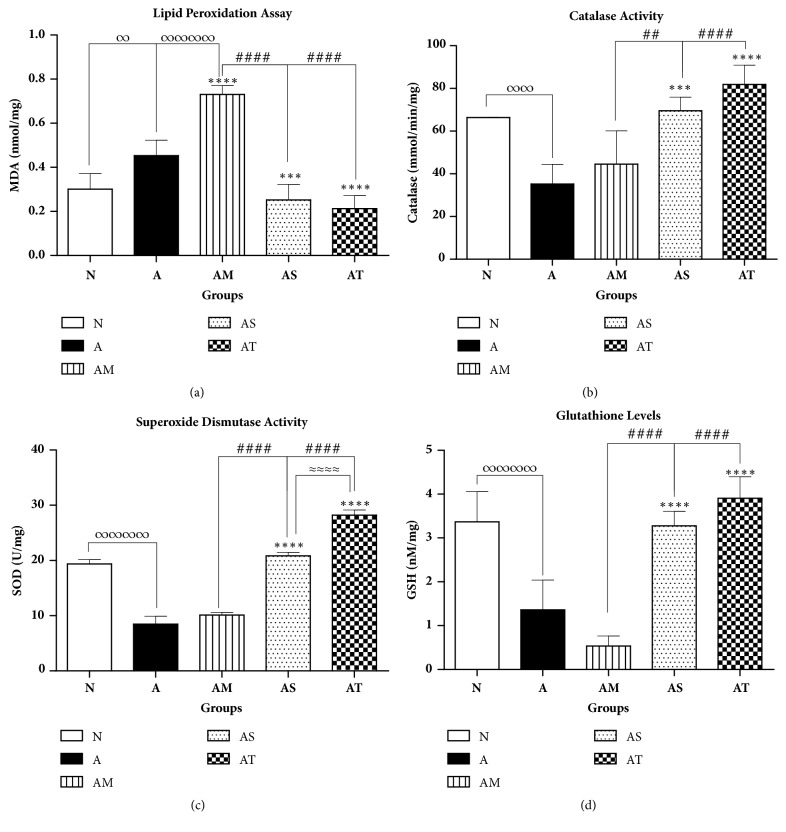
**Effect of treatments on liver oxidative stress and antioxidative defense biomarkers**. (a) Lipid peroxidation levels (MDA); enzymatic activities of (b) catalase, (c) SOD, and (d) glutathione levels liver homogenates. All values are presented as mean ± SD of 8 rats per group. ∞p< 0.05, ∞∞p<0.01, and ∞∞∞∞p<0.0001 for A and/or AM compared with N. *∗∗∗*p<0.001, *∗∗∗∗*p<0.0001 compared with arthritic group A. ##p<0.01, ####p<0.0001 compared with positive control AM. ≈≈≈≈p<0.0001 between AS and AT.

Untreated AIA group exhibited a significant increase in mean MDA levels when compared to normal group, reflecting the occurrence of oxidative stress in arthritic rats. Weekly treatment of MTX resulted in further increase in MDA levels in liver homogenates as compared to untreated AIA group, indicating the presence of hepatotoxicity associated with MTX usage. In contrast, treatment with sesame oil or Δ9-THC/sesame oil reduced significantly mean MDA values when compared to untreated AIA group (by 44% and 53%, respectively) and MTX-treated group (by 66% and 71%, respectively) with no statistical difference relative to the normal group (Figure 6(a)).

As for the antioxidative defense system, our results revealed a reduced antioxidative defense system associated with AIA. Catalase and SOD activities and GSH levels were found to be reduced in untreated AIA group by 65%, 60%, and 56%, respectively, when compared to the normal group. Treatment with MTX did not improve the situation. In contrast, the antioxidative enzymatic activities of catalase and SOD and the levels of GSH were markedly suppressed in this group (by 45%, 48%, and 84%, respectively) compared to the normal one.

Treatment with sesame oil or Δ9-THC/sesame oil significantly improved the activities of catalase and SOD and markedly increased the levels of GSH when compared to untreated AIA group and MTX-treated group, with no statistical difference relative to the normal group. Moreover, SOD activity in Δ9-THC/sesame oil-treated group were found to be significantly higher than those in sesame oil-treated AIA group, suggesting a better antioxidative activity of Δ9-THC on liver tissue (Figure 6(c)).

## 4. Discussion

To our knowledge, this is the first study to present a potential therapeutic value of Dronabinol (Δ9-THC/sesame oil) on RA by demonstrating its anti-inflammatory and antiarthritic effects on AIA rat model and its protective impact on the oxidative stress produced by the disease itself. Earlier studies have presented the immune-modulating effect of endocannabinoids on autoimmune diseases such as RA, suggesting a potential role for exogenous cannabinoids as therapeutic agents for this disease [40, 41].

Arthritis was induced by CFA injections in the left hind paw. CFA-induced arthritis is characterized by the production of a strong and long-lasting inflammatory response at the site of injection within days after its administration [42]. This model was successfully manipulated in our study and used to mimic RA in humans at the level of clinical symptoms, histological and immunological aspects. The disease symptoms were manifested in the untreated AIA group whose rats suffered from severe erythema and edema at the site of CFA injection. Moreover, H&E staining of joint sections revealed increase in synoviocytes and hyperplasia of the intimal lining layer, cellular infiltration, pannus formation, and cartilage erosion in AIA rats. Systemic inflammation was also reflected in high levels of ESR in addition to arising values of proinflammatory cytokines (IL-6, IL-1*β*, and TNF-*α*) in AIA rat sera. These cytokines are usually abundant in RA and act as key mediators of cell migration and inflammation. Their local effects on the joint include recruitment of neutrophils and activation of osteoclasts, chondrocytes, and synoviocytes. Neutrophils contribute to inflammation and joint destruction by secreting proteolytic enzymes and reactive oxygen intermediates. Osteoclasts, chondrocytes, and synoviocytes are involved in cytokine-mediated angiogenesis that will eventually lead to pannus formation and cartilage and bone destruction [3, 43].

Stressing on the significant role of oxidative stress in RA pathogenesis, our study revealed a deficiency in the antioxidative defense system and an increase in the prooxidant markers. This was clear in the arthritic rat group that showed declined levels in the enzymatic antioxidant activities of SOD and catalase and the nonenzymatic antioxidant levels of GSH, yet, intensified levels of lipid peroxidation. Our results come in agreement with other studies that showed the implication of oxidative stress in RA pathogenesis [44–48].

MTX was used as positive control in our experiment where it presented similar antiarthritic effects as recorded by others [49, 50]. Being a folic acid antagonist, MTX blocks the synthesis of purines and pyrimidines by inhibiting key enzymes, which eventually attenuates DNA/protein/lipid methylation and blocks the de novo purine synthesis. It also evokes adenosine release leading to adenosine-mediated immunosuppression. These, in addition to other pharmacological mechanisms of action, are thought to be the underlying causes for the anti-inflammatory effect of MTX and, thus, for its effectiveness in RA treatment [12]. However, its association with several side effects, especially hepatotoxicity, necessitates reconsidering MTX for long-term treatment of RA [11]. This toxicity was well demonstrated in our study in MTX-treated AIA rats, and our findings are in line with previous reports showing damage in the liver tissue following MTX therapy [13, 51–53]. Thus, seeking natural alternatives, having less drawbacks, is recommended for RA treatment especially on the long term, and many natural compounds have manifested promising results as therapeutic agents in this field [54].

Several clinical and preclinical studies have demonstrated that cannabis-derived drugs constitute a potent treatment modality against inflammatory disorders, such as RA [55–57]. In fact, cannabinoids were found to exert immunosuppressive functions through inducing apoptosis of immune cells, downregulating cytokine and chemokine production, and upregulating regulatory T cells [58]. For instance, Δ9-THC was shown to inhibit macrophage activity, thus, diminishing the release of proinflammatory cytokines and attenuating inflammation [59, 60]. This can explain the reduced values of these cytokines in Δ9-THC/sesame oil-treated group. Δ9-THC was also demonstrated to inhibit the transcription of inducible nitric oxide synthase (iNOS) and nitric oxide (NO) production in response to LPS. Overproduction of NO is a key factor in RA pathogenesis [61].

In the context of RA, a significant finding regarding the endocannabinoid system, specifically CB1 and CB2 receptors, was found in RA and osteoarthritic patients who had high expression levels of these receptors in their synovium compared to controls [62]. This adds another hint for the possible role of this system in RA pathogenesis and for the potential use of synthetic cannabinoids to target RA, especially that the immune-modulatory effects of these cannabinoids are mediated through cannabinoid receptors, mainly CB2 [63].

Cannabidiol was tested* in vivo* and showed prominent results as anti-inflammatory and antioxidant agent for RA [64]. However, little, if any, research has been done on Δ9-THC in animal models of RA.

Employing antioxidants is an added value in RA treatment. Sesame oil exhibits potential anti-inflammatory and antioxidative effects [26–29]. Thus, Dronabinol was used as a treatment, since it is composed of the synthetic Δ9-THC dissolved in sesame oil.

Our results for Dronabinol are considered the first to be recorded in RA experimental model. Its effectiveness can be compared to that of MTX. In Dronabinol-treated AIA rats, we witnessed, after 21 days of treatment, results that were in several aspects similar to normal rats; these include proinflammatory cytokines and ESR levels. As for mean body weight change during the treatment, the improvement presented in sesame oil- and Δ9-THC/sesame oil-treated group might be attributed to the biochemical fatty composition of sesame oil and its contribution to increasing body weight [65]. Thus, we cannot rely on body weight results to advocate the alleviating roles of sesame oil or Dronabinol in RA; instead, we focused on macroscopic mean scores and inflammatory markers.

Sesame oil diminished the levels of proinflammatory cytokines. This comes in agreement with a previous study [27], where minor components of sesame oil had similar impact on these cytokines. This anti-inflammatory effect was also demonstrated to be mediated by suppression of iNOS production and inhibition of neutrophil infiltration [66].

The main advantage of sesame oil and Δ9-THC over MTX lies in the hepatoprotective effect against the oxidative stress induced by the disease. Histological examination of liver sections assured the presence of damage due to MTX treatment as reflected by severe portal and lobular inflammation, neutrophil infiltration, vascular angiogenesis, and multinucleation. These manifestations were minimal to absent in sesame oil- and Δ9-THC/sesame oil-treated AIA rats, respectively. Indeed, Δ9-THC/sesame oil treatment resulted in the most pronounced effect. Elevated antioxidative enzyme activity compared with decreased lipid peroxidation levels and relatively normal liver histology demonstrated the antioxidative effect of this combination.

Our results are consistent with other studies proving an antioxidative effect of sesame oil in arthritic rats [26, 27, 29, 67] and of Δ9-THC in some* in vitro* and* in vivo* models [68–70]. Despite the remarkable effects of sesame oil monotherapy in arthritic rats, Δ9-THC/sesame oil exhibited slightly better antiarthritic and anti-inflammatory effects. Indeed, Δ9-THC/sesame oil restored the normal architecture of the left hind paw in arthritic rats similarly to the control group and induced a significant increase in SOD levels compared to sesame oil alone. Moreover, Δ9-THC/sesame oil therapy was well tolerated in arthritic rats with no particular psychoactive side effects at the dose used.

Dronabinol (Δ9-THC in sesame oil) is usually used to treat nausea and vomiting caused by chemotherapy or weight loss and loss of appetite in AIDS patients, yet, to the best of our knowledge, this is the first study that proves the antiarthritic and antioxidative effects of this combination in an experimental model of RA with a hepatoprotective effect against arthritis-induced liver injury compared to commonly used antirheumatic drug (MTX).

Future studies that investigate the precise mechanism of action of Dronabinol in AIA model and test the possible analgesic effect this drug are recommended to encompass wider manifestations of the disease including pain, which is one of the major sufferings of RA patients that is not targeted by MTX.

## Data Availability

The data underlying the findings of this article can be provided by the corresponding author upon request.
